# Personalized Medicine Interventions for Glycemic Control in Adults With Type 2 Diabetes: A Systematic Review

**DOI:** 10.7759/cureus.106693

**Published:** 2026-04-09

**Authors:** Hadeil Elfadil Ahmed Abdelgader, Shimaa Ali Hassan Hamed, Hamza Abubaker Abdin Sidahmed, Ahmed-Lamin Gehani, Abdulwahhab Al-Shaikhli, Amani Edris Uthman Ali, Hiba Merghani Abdalkareem Hajali, Elkhansaa A Elsamani, Asim Ahmed

**Affiliations:** 1 Medicine, Hamad Medical Corporation (HMC), Doha, QAT; 2 Internal Medicine, University of Bahri, Khartoum, SDN; 3 Emergency Medicine, Al Qadarif Teaching Hospital, Al Qadarif, SDN; 4 Medical Claims, Almadallah Healthcare Management, Dubai, ARE; 5 Medicine, Near East University, Lefkosa, CYP; 6 General Practice/Pediatrics, Gardenia Medical Center, Doha, QAT; 7 Umm Ghuwailina (Doha Department), Primary Health Care Corporation, Doha, QAT; 8 Medicine, Omdurman Islamic University, Abudhabi, ARE; 9 Internal Medicine, Royal Care International Hospital, Khartoum, SDN; 10 Medicine and Surgery, University of Gezira, Riyadh, SDN

**Keywords:** continuous glucose monitoring, digital health, glycemic control, personalized medicine, type2 diabetes mellitus

## Abstract

Type 2 diabetes mellitus (T2DM) is a heterogeneous condition in which patients differ markedly in glycemic patterns, treatment responses, and self-management capacity, limiting the effectiveness of uniform care models. Personalized medicine aims to tailor diabetes management using patient-specific data, behaviors, and risk profiles, but the clinical impact of different personalization strategies remains variable. A comprehensive search of major biomedical databases was conducted to identify eligible studies evaluating individualized interventions in adults with T2DM, and 27 studies were included. Evidence was synthesized narratively due to clinical and methodological heterogeneity. Across the included studies, personalized approaches were generally associated with improved glycemic control compared with usual care, although the magnitude and consistency of benefit varied by intervention type and implementation intensity. Larger and more durable improvements were most often observed in interventions that combined objective patient data with actionable care pathways, particularly in high-risk populations or during periods of therapeutic change. These findings suggest that personalization may improve glycemic outcomes in certain settings, although the certainty of evidence remains limited due to study heterogeneity and variations in design and quality.

## Introduction and background

Type 2 diabetes mellitus (T2DM) presents uniquely in each person, with variations in blood sugar patterns and treatment outcomes influenced by behavior, comorbidities, and self-management capacity. Because of this heterogeneity, a uniform approach to care is often inadequate. Personalized care can be defined as adapting management to the individual by using patient-specific data to modify self-management support, follow-up intensity, and or therapy. In this review, personalized medicine refers to interventions that adapt diabetes management using patient-level clinical, behavioral, or glucose-related data to guide treatment selection, monitoring, support intensity, or care pathways. Standard care, in contrast, refers to conventional guideline-based management delivered with minimal customization to individual patient data and circumstances. Computerized self-management programs that provide structured, individualized guidance have been associated with improved glycemic control in adults with T2DM [1]. However, across the literature, uncertainty remains about which components of personalization are essential for durable benefit and how consistently these interventions translate into clinically meaningful improvements across settings [2].

Digital tools increasingly enable personalized care through tailored education, reminders, and feedback. Evidence syntheses suggest that digital self-management interventions can improve HbA1c, although effects vary with intervention intensity, the degree of tailoring, and sustained engagement [2]. Telemedicine and remote monitoring can further enhance personalization by enabling ongoing review of patient data and timely feedback, with stronger performance when embedded into clinical workflows and designed to trigger actionable responses rather than passive observation [3]. Their real-world impact, however, also depends on implementation barriers, including workflow integration demands and differences in patient capacity to use digital tools effectively [3].

Diabetes education and support platforms appear most effective when they adapt to behavioral patterns, biometric signals, and predefined risk categories over time, rather than relying only on static information or fixed glycemic thresholds [4]. In practice, adaptability may be operationalized through feedback loops that use patient data to adjust content, prompts, and escalation pathways over time, supporting targeted support for those at higher risk or with worsening control [4]. Beyond technology, personalized approaches may also be delivered through health system redesign and strengthened follow-up, including in resource-limited settings where feasibility and scalability are central [5].

Overall, current evidence shows personalized strategies can improve glycemic control. However, effects differ by intervention type, intensity, patient engagement, and the extent to which patient-specific data inform clinical decisions [2]. Variability in baseline risk, treatment context, and individual patient factors may further modify responses [5]. Despite these insights, the literature remains heterogeneous, making it unclear which forms of personalization are most effective, which patient groups benefit most, and whether observed improvements reflect true tailoring of care, greater intervention intensity, or both. These uncertainties justify a systematic review to determine which personalized approaches reliably reduce HbA1c, for which patients, and under what implementation conditions.

This review aims to systematically identify and synthesize evidence on personalized medicine interventions for improving glycemic control in adults with T2DM, compared with standard or non-personalized care. The primary objective is to evaluate the effect of personalized interventions on glycemic control using HbA1c as the primary measure, where reported. Secondary objectives are to compare effectiveness across personalized intervention types (e.g., digitally tailored support, individualized lifestyle programs, and other data-driven approaches) versus standard care; to assess whether patient characteristics (e.g., age, lifestyle, comorbidities, and other individual factors) modify response; and to synthesize additional outcomes including fasting glucose, insulin sensitivity/insulin resistance, weight, medication adherence or adjustment, and cost-effectiveness where available.

Literature review

Patient-centered self-management approaches align diabetes care with individual needs and preferences and have demonstrated improvements in glycemic outcomes compared with usual care [6]. Pharmacist-led personalization can help optimize medication management and support adherence in routine practice. Systematic evidence indicates HbA1c benefit, with effect sizes varying across settings and intervention intensity [7,8]. Variation in setting, patient case mix, baseline glycemic control, and implementation fidelity likely explains part of the between-study variability [7,8].

For people with persistently poor control in primary care, multicomponent interventions that combine structured follow-up with treatment adjustment are more effective than single-component approaches [9]. Patient activation strategies may further strengthen sustained self-management behaviors and improve glycemic outcomes, particularly when follow-up is ongoing [10].

Data-driven personalization using continuous glucose monitoring (CGM) supports individualized behavioral and therapeutic adjustments by providing high-resolution glucose profiles. Earlier meta-analyses suggest CGM can improve glycemic outcomes under certain conditions [11]. Later syntheses focusing on T2DM support improvements in HbA1c, particularly when CGM data are translated into structured coaching or decision pathways [12]. Intermittent CGM scanning may also help, especially when paired with education or support that helps patients act on glucose patterns [13]. Randomized evidence in basal insulin-treated T2DM shows clinically meaningful HbA1c improvement with CGM versus standard monitoring, reinforcing CGM-enabled personalization as a practical outpatient strategy [14]. Taken together, these prior studies and evidence syntheses suggest that digital self-management support, CGM-informed care, and pharmacist-led interventions are among the most established pathways through which personalization may improve glycemic outcomes in adults with T2DM, although uncertainty remains regarding comparative effectiveness and durability across settings.

## Review

Methodology

Design and Reporting

This systematic review was conducted and reported in accordance with Preferred Reporting Items for Systematic Reviews and Meta-Analyses (PRISMA) 2020 guidance [15]. No protocol was prospectively registered. A narrative synthesis approach was used because substantial clinical and methodological heterogeneity was anticipated across personalization mechanisms, e.g., digital tailoring, telemonitoring workflows, CGM-informed pathways, intervention intensity, and outcome definitions and timepoints.

Eligibility Criteria

Eligibility was defined using the PICOS framework (Table 1).

**Table 1:** PICOS eligibility criteria for study selection. PICOS: Population, Intervention, Comparator, Outcomes, Study design. The table summarizes the predefined inclusion criteria used to screen and select eligible studies for this systematic review. RCT, randomized controlled trial; CGM, continuous glucose monitoring; QoL, quality of life; BMI, body mass index

Component	Definition for this review
Population (P)	Adults (≥18 years) with type 2 diabetes mellitus (any care setting)
Intervention (I)	Personalized/individualized interventions explicitly tailored using patient-level characteristics or data (e.g., CGM-informed coaching/decision pathways; tailored digital/app programs; telemonitoring with individualized feedback/escalation rules; pharmacist-led individualized medication optimization; tailored patient activation/person-centered programs; tailored care-pathway/system interventions)
Comparator (C)	Usual care, guideline-based care, minimal care, or a non-personalized version of the intervention
Outcomes (O)	Primary: HbA1c. Secondary: Fasting glucose; CGM metrics (e.g., time-in-range/variability); weight/BMI; medication adherence and/or treatment intensification; hypoglycemia; patient-reported outcomes (e.g., QoL, distress, self-efficacy); costs/cost-effectiveness (if reported)
Study designs (S)	RCTs or cluster RCTs, quasi-experimental controlled studies, prospective or retrospective comparative cohort studies, and pre-post interventional or quality improvement studies; English; 2005 to 2025

Studies were eligible if they included adults ≥18 years with T2DM and evaluated a personalized or individualized intervention explicitly tailored using patient level data or characteristics, e.g., CGM-informed coaching or decision pathways, tailored digital or app programs, telemonitoring with individualized feedback or escalation rules, pharmacist-led individualized medication optimization, tailored patient activation or person-centered programs, or tailored care pathway or system interventions. For studies that included mixed diabetes populations, data were extracted only when outcomes for adults with T2DM were reported separately. Comparators included usual care, guideline-based care, minimal care, or a non-personalized version of the intervention. The primary outcome was HbA1c; secondary outcomes included fasting glucose, CGM metrics (e.g., time in range), weight or BMI, medication adherence or treatment intensification, hypoglycemia, patient-reported outcomes, and cost or cost-effectiveness, when available.

Eligible designs included randomized controlled trials (RCTs) or cluster RCTs, quasi-experimental controlled studies, prospective or retrospective comparative cohort studies, and pre-post interventional or quality improvement studies. Limits were English-language publications from 2005 to 2025.

Studies were excluded if they involved non-adult populations, did not report T2DM data separately, lacked a personalized intervention component, were non-comparative or ineligible by design, or were available only as conference abstracts without a full, eligible journal report.

Information Sources and Search Strategy

A comprehensive search was conducted in PubMed/MEDLINE, Embase, Cochrane CENTRAL, CINAHL, Scopus, and Web of Science, with Google Scholar used as a supplementary source for sensitivity searching and citation chasing (Table 2).

**Table 2:** Database search strategy framework and key terms. The table outlines the core search concepts and example keyword structures used across databases. Search syntax was adapted to each database while retaining the same conceptual structure: T2DM + personalization + enabling modality + glycemic outcomes. The full final search strings for each database, along with database-specific execution limits, are provided in the Appendix.

Database	Limits	Core concepts and example terms
PubMed/MEDLINE	2005-2025; Humans; English; Adults	T2DM terms AND personalized OR precision OR individualized OR tailored OR stratified OR patient-centered OR patient activation AND telemedicine OR telemonitoring OR digital health OR mHealth OR app OR decision support OR EHR OR CGM OR flash glucose monitoring AND HbA1c OR glycemic control
Embase	2005-2025; English; Adults	Emtree plus keywords for T2DM AND personalization or tailoring AND telehealth or digital or CGM or decision support AND HbA1c or glycemia using proximity operators
Cochrane CENTRAL	Search broadly; apply limits in screening	T2DM AND (personalized OR tailored OR precision OR CGM OR telemonitoring OR telehealth OR digital OR decision support) AND (HbA1c OR glycemic)
CINAHL	2005-2025; English; Adults	T2DM AND (tailored/personalized OR patient-centered OR activation OR telehealth OR digital OR app OR CGM) AND HbA1c/glycemic outcomes
Scopus/Web of Science	2005-2025; English	TITLE/ABSTRACT/KEYWORDS: T2DM AND (personalization/tailoring OR CGM OR telehealth OR digital OR decision support) AND HbA1c/glycemic control
Google Scholar (supplementary)	2005-2025; English	“type 2 diabetes” AND (personalized OR tailored OR precision OR CGM OR telehealth OR telemonitoring OR “digital health”) AND (HbA1c OR glycemic control)

Search concepts combined were T2DM, personalization, personalized, precision, individualized or tailored or stratified, enabling modalities digital health, telemedicine, telemonitoring, CGM or flash glucose monitoring, and decision support or electronic health record (EHR), and glycemic outcomes HbA1c or glycemic control. Search syntax was adapted for each database. The full final search strings for each database, the date of the final search, and the database-specific execution limits are provided in the Appendix.

Study Selection

All retrieved records were imported into EndNote X9 Clarivate Plc, London, United Kingdom, and duplicates were removed. Two reviewers independently screened titles and abstracts, followed by full-text assessment of potentially eligible articles. Before full screening, a pilot exercise with 50 records was conducted to refine and calibrate the criteria, ensuring consistency and methodological rigor. Disagreements were resolved through discussion or by consulting a senior reviewer.

Data Extraction

Two reviewers independently extracted data using a piloted extraction form. Extracted items included study design and setting, sample size and participant characteristics, baseline HbA1c and treatment regimen, intervention description and personalization signal, comparator details, delivery model and follow-up duration, outcome definitions and timepoints, key results, including glycemic and adherence metrics, co-interventions, and implementation features, such as workflow integration, staffing, and technology requirements.

Risk-of-Bias Assessment

Risk of bias was assessed independently by two reviewers. Randomized trials were evaluated using the Cochrane Risk of Bias 2 tool (RoB 2), including cluster and crossover variants, as applicable [16]. Nonrandomized comparative studies were assessed using the ROBINS-I tool [17]. Because ROBINS-I is primarily intended for nonrandomized studies with a comparator, the single quality improvement pre-post study without a concurrent comparator was not judged as directly comparable to the controlled studies and was interpreted separately with caution as high uncertainty evidence. Disagreements were resolved through discussion and consensus or adjudication by a third reviewer. Risk-of-bias judgments were summarized using domain-level tables for randomized trials and a ROBINS-I-informed traffic light plot for nonrandomized studies.

Data Synthesis

Findings were synthesized narratively and grouped a priori by personalization type, e.g., tailored digital or app-based programs, telemonitoring or telehealth feedback models, CGM-informed coaching or decision pathways, pharmacist-led individualized medication optimization, patient activation or person-centered tailoring, and tailored care pathway or system interventions. For HbA1c, results were summarized by direction and magnitude of effect as reported and by follow up timepoint. Secondary outcomes were summarized in parallel where reported. Where available, effect modifiers baseline HbA1c, insulin use, engagement level, and care setting were extracted and synthesized narratively. Because the included studies differed in design, baseline HbA1c, intervention intensity, and effect measure type, effect sizes were interpreted descriptively and were not considered directly comparable across studies or intervention categories.

Strengths

This review used a predefined PICOS framework and a multi-database search strategy to maximize capture of relevant personalization approaches. Dual independent screening, extraction, and risk of bias assessment reduced selection and appraisal bias. Grouping results by personalization type and extracting implementation and engagement features improved interpretability and practical relevance for real-world adoption.

Results

Study Selection

**Figure 1:**
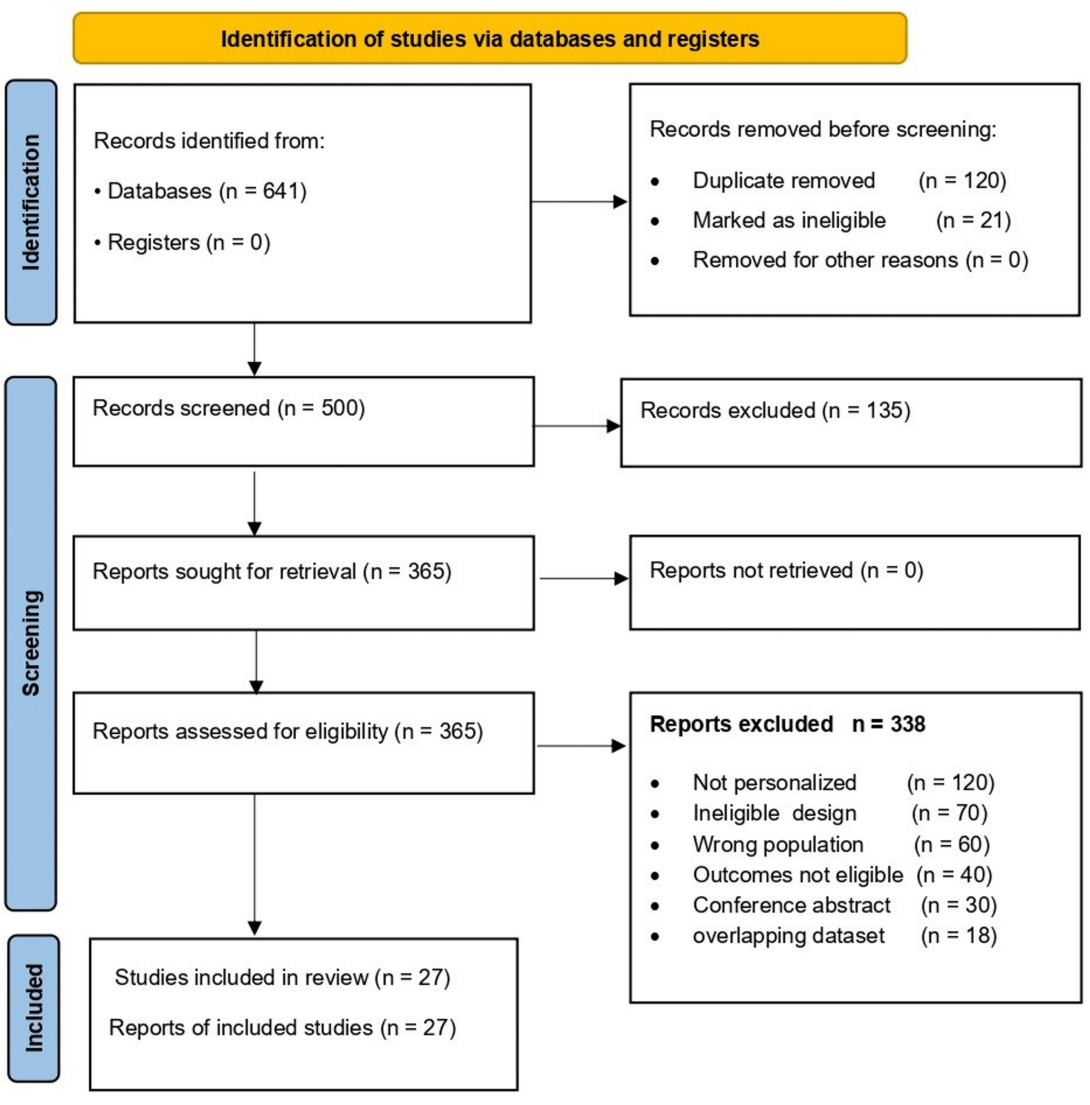
PRISMA 2020 flow diagram of study selection. Flow diagram illustrating the identification, screening, eligibility assessment, and inclusion of studies in this systematic review. Records were identified from databases and screened after the removal of duplicates and ineligible records. Full-text articles were assessed for eligibility, with reasons for exclusion summarized at the eligibility stage. The final number of studies included in the qualitative (narrative) synthesis is shown. PRISMA, Preferred Reporting Items for Systematic Reviews and Meta-Analyses

A total of 641 records were identified through database searching. After removing 120 duplicates and 21 records marked as ineligible by automation tools, 500 records remained for title and abstract screening. During screening, 135 records were excluded, and 365 reports were sought for retrieval. All reports were successfully retrieved, and 365 full-text articles were assessed for eligibility. Of these, 338 full-text articles were excluded. A total of 27 studies were included in the qualitative narrative synthesis. Conference abstracts were excluded unless a full peer-reviewed journal article reporting the same study was available and eligible. All studies included in the final synthesis were published as full journal articles (Figure 1).

Overview of Included Evidence

A total of 27 studies [18-44] assessing personalized medicine interventions for adults with T2DM were synthesized.

In this review, personalized medicine refers broadly to patient-tailored care strategies that adapt management using individual clinical data, behavioral information, or treatment response rather than genomic precision medicine alone. Interventions were grouped into: (1) digitally delivered personalized coaching, telehealth support, and remote monitoring systems; (2) CGM-enabled personalization and feedback systems; (3) individualized lifestyle and nutrition programs; (4) pharmacist-led or collaborative care; (5) decision support or algorithm-based treatment selection; and (6) stratified pharmacotherapy and economic stratification frameworks. The primary glycemic outcome was HbA1c. Several studies also reported CGM-derived metrics such as time in range [19,34,37], adherence outcomes [23], peak glucose [26], cost-effectiveness measures [27], and patient-reported outcomes, including distress, self-efficacy, and quality of life [32,44].

Study-Level Results

Table 3 presents the key findings of all included studies, organized by study.

**Table 3:** Included studies and key outcomes (n = 27). RCT, randomized controlled trial; QI, quality improvement; CGM, continuous glucose monitoring; TIR, time in range; EMR, electronic medical record; SBP, systolic blood pressure; DBP, diastolic blood pressure; ED, emergency department; BMI, body mass index; FBG, fasting blood glucose; ICER, incremental cost-effectiveness ratio; QALY, quality-adjusted life year; UC, usual care; CI, confidence interval; ITT, intention-to-treat; PDS, personalized decision support; iPDM, integrated personalized diabetes management; GEM, minimizing glucose excursions method; eGFR, estimated glomerular filtration rate; FMD, flow-mediated dilation; NR, not reported

Study ID [Ref]	Design	Sample size (*n*)	Personalized intervention	Comparator	HbA1c outcome (as reported)	Other outcomes
Lauffenburger et al. (2019) [18]	RCT (three-arm pragmatic)	6,000	Risk/need stratified intensity	Three-arm comparison	Arm 3 vs. 1: –0.25% (95% CI –0.43 to -0.06); Arm 2 vs. 1: -0.15%	Similar spending; Arm 2 had more hospitalizations/ED visits.
Simonson et al. (2021) [19]	QI (pre-post)	68	Professional CGM + counseling	Pre-post	8.8% ±1.2 → 8.2% ±1.3 (*P* = 0.006)	Improved TIR; more hypoglycemia in the subset.
Varming et al. (2019) [20]	RCT (unblinded)	97	Patient-centered goal-setting	Usual care	No superiority	Increased autonomy support and healthy eating.
Milani et al. (2021) [21]	Cohort (matched)	763 vs. 794	Digital medicine program	Usual care	7.3% → 6.9% (P < 0.001)	HbA1c ≥9% decreased; distress and hypoglycemia reduced.
Lee et al. (2018) [22]	RCT	148	Tailored mobile coaching	Usual care	HbA1c decreased from 8.1 ± 1.4% to 7.5 ± 1.1% at 6 months in the intervention group	Improved frequency of blood glucose testing and exercise.
Frias et al. (2017) [23]	Cluster RCT	109	Digital medicine offering (DMO)	Usual care	Favored DMO (magnitude NR)	Greater SBP reduction (-21.8 vs. -12.7 mmHg); adherence ≥80%.
Rein et al. (2022) [24]	Randomized crossover pilot	23 (XO); 16 (Ext)	ML-guided postprandial diet	Mediterranean diet	-0.39% (Extension phase)	Better CGM metrics; 61% remission (HbA1c <6.5%).
Bertsimas et al. (2017) [25]	Retrospective EMR	10,806	Algorithm-based treatment	Standard care	7.93% vs. 8.37% (P < 0.001)	Algorithm matched standard care in 68.2% of visits.
Chang et al. (2025) [26]	RCT (waitlist)	NR	CGM-timed exercise	Control	No intervention effect	24-h peak glucose decreased; adherence >90%.
Slingerland et al. (2013) [27]	Cluster RCT	506	Stratified patient-centered care	Usual care	HbA1c >8.5%: -0.83% at 1 year	High stratum: ICER 261 USD/QALY; low stratum not cost-effective.
Javaid et al. (2019) [28]	Open-label RCT	244	Pharmacist-led collab care	Control	10.9% → 7.7% vs. control 10.3% → 9.7%	Improved SBP, DBP, and triglycerides.
Ramallo-Fariña et al. (2020) [29]	Multiarm cluster RCT	2,334	Multi-level (patient/prof)	Usual care	Patient arm: -0.27% (3m) and -0.26% (6m)	Uncontrolled baseline patients improved across all arms.
Griauzde et al. (2022) [30]	Pragmatic RCT (QI)	1,584	Enhanced care (CGM + coaching)	Usual care	-0.41% diff vs. UC (*P* = 0.04)	CGM users had 1.1% lower HbA1c vs. baseline.
Lee et al. (2022) [31]	Three-arm RCT	269	Mobile personalized feedback	MC and UC	12 wks: -1.04% (MPC) vs. -0.49% (UC)	No diff at 26 wks; larger benefit in age <65/BMI ≥25.
Crowley et al. (2022) [32]	Active-comparator RCT	200	Comprehensive telehealth	Telemonitoring	-1.59% vs. -0.98% (*P* = 0.02)	Improved distress, self-care, and self-efficacy.
Wong (2023) [33]	Retrospective cohort	180	CGM-guided intervention	Control	Adjusted diff: -0.40% (*P* = 0.005)	High rates of medication and dietary adjustment.
Martens et al. (2025) [34]	RCT (6 months)	72	CGM ± food app	Within-study	Overall reduction: -1.3% (P < 0.001)	TIR increased 46%→72%; weight loss of 7 lb.
Halalau et al. (2022) [35]	Open-label RCT	86	Pharmacist-managed care	Control	-2.85% vs. -1.32% (*P* = 0.005)	3.15× higher odds of HbA1c ≤8%.
Li et al. (2025) [36]	Prospective comparative	120	Internet remote management	Traditional care	6.5% vs. 7.2% (P < 0.001)	Remission: 60% vs. 37%; improved β-cell function.
Hangaard et al. (2025) [37]	Open-label RCT	331	Telemonitoring	Standard care	NR	CGM TIR improved (+13.6%, *P* = 0.004).
Caballero Mateos et al. (2025) [38]	Multicenter RCT	85	Personalized education	Control	3.7% vs. 2.6% reduction (*P* = 0.006)	Greater weight/BMI reduction and knowledge.
Bersch-Ferreira et al. (2024) [39]	Open-label RCT	371	Nutritional strategy	Control	No between-group difference	Similar glycemic control achievement (~19%).
Augstein et al. (2010) [40]	Retrospective obs	289	Personalized decision support	Usual behavior	7.10% → 6.73%	Nonuse linked to HbA1c increase.
Romadlon et al. (2025) [41]	Three-arm RCT	84	Texting ± peer support	Prof education	Reduced vs. education (mag. NR)	Remission: 50% (peer) vs. 25% (text) vs. 3.7% (edu)
Kulzer et al. (2018) [42]	Cluster RCT	907	Integrated mgmt (iPDM)	Usual care	-0.5% vs. -0.3% (*P* = 0.03)	More therapy adjustments; higher satisfaction
Shields et al. (2023) [43]	Three-way crossover RCT	525	Stratified pharmacotherapy	Cross-drug	Overall HbA1c similar	BMI >30: pioglitazone better; eGFR 60-90: sitagliptin better
Cox et al. (2020) [44]	RCT (2:1)	30	GEM-CGM program	Routine care	8.9% → 7.6% vs. 8.8% → 8.7%	Reduced meds/carbs; improved QoL/distress

Synthesis of HbA1c Effects Across Intervention Types

Overall, HbA1c improvements ranged from modest (~0.2%-0.4%) to large (≥1.0-2.85%), with larger effects typically observed when interventions were intensive, targeted to high-risk groups, or delivered via multidisciplinary or pharmacist-led models. To support interpretability, HbA1c findings were synthesized by intervention class (Table 4).

**Table 4:** HbA1c outcomes by intervention category. Overall, digital and CGM-based interventions were frequently associated with HbA1c improvement, while pharmacist-led collaborative care and intensive remote management showed substantial benefit in some studies, particularly among patients with high baseline HbA1c [21,28,32,35]. However, these patterns should be interpreted cautiously because the included studies differed in baseline glycemic status, effect measure type, study design, and risk of bias [21,30,31,32]. HbA1c, glycated hemoglobin; CGM, continuous glucose monitoring; TIR, time in range; FMD, flow-mediated dilation; PDS, personalized decision support; BMI, body mass index; eGFR, estimated glomerular filtration rate.

Intervention category	Evidence pattern (based on included studies)	Illustrative findings (as reported)
Digital coaching/Telehealth/Remote monitoring	HbA1c generally improved compared with usual care; larger reductions were observed in more comprehensive programs and in cohorts with poorer baseline control.	Milani et al. [21]: 7.3 → 6.9 vs. no change; Crowley et al. [32]: –1.59 vs. -0.98; Li et al. [36]: 6.5 vs. 7.2
CGM-enabled personalization and feedback	HbA1c reductions were commonly reported; effect size varied by engagement and/or CGM uptake.	Simonson et al. [19]: 8.8 → 8.2; Wong [33]: -0.40; Martens et al. [34]: -1.3; Cox et al. [44]: 8.9 → 7.6; Griauzde et al. [30]: Larger improvement among CGM users
Individualized lifestyle/Nutrition	Effects were heterogeneous: clinically meaningful improvements were reported in some algorithm-guided or structured programs, while others showed neutral HbA1c effects.	Rein et al. [24]: -0.39 and 61% remission; Augstein et al. [40]: 7.10 → 6.73 with PDS use; Chang et al. [26]: No HbA1c change (peak glucose/FMD improved); Varming et al. [20]: No superiority
Pharmacist-led/Collaborative care	These models showed the largest HbA1c reductions, particularly in studies enrolling participants with high baseline HbA1c.	Javaid et al. [28]: 10.9 → 7.7 vs. control 10.3 → 9.7; Halalau et al. [35]: -2.85 vs. -1.32
Decision-support/Algorithmic selection	Modest but statistically significant HbA1c advantages were reported in selected settings.	Bertsimas et al. [25]: 0.44% lower HbA1c vs. standard decisions; Lauffenburger et al. [18] Arm 3: -0.25% difference vs. low-intensity
Precision stratification (clinical markers)	Overall HbA1c was similar across drug classes; however, clinically relevant subgroup differences were reported.	Shields et al. [43]: Differential responses by BMI and eGFR strata

Digital Coaching Versus Usual Care

Across studies, digital coaching and telehealth interventions often improved HbA1c compared with usual care, particularly when programs were comprehensive and or targeted to patients with persistently uncontrolled diabetes. Milani et al. [21] reported a reduction in HbA1c from 7.3% to 6.9%, while usual care showed no change. Crowley et al. [32] reported greater HbA1c improvement with comprehensive telehealth than with a more straightforward telemonitoring approach. However, some digital feedback models showed early benefits that were not sustained over longer follow-up [31], and engagement levels were a major driver of observed effectiveness [30].

Cost-Effectiveness and Stratified Allocation of Intensive Care

Economic evidence supported the principle that personalization is most efficient when concentrated in higher-risk strata. Stratified patient-centered care demonstrated the strongest cost-effectiveness among patients with baseline HbA1c >8.5%, with an incremental cost-effectiveness ratio (ICER) of USD 261/QALY and an HbA1c reduction of 0.83% at one year [27]. In contrast, stratified care was not cost-effective for individuals with baseline HbA1c <7.0% [27]. In persistently poorly controlled diabetes, comprehensive telehealth improved HbA1c by 1.59% at an additional annual cost of USD 1,519 per patient, with this cost interpreted in the context of the observed clinical benefit and the targeted high-risk population [32].

Summary of Primary Outcome

Across 27 studies, personalized medicine interventions were frequently associated with improved glycemic control as measured by HbA1c. Improvement was reported in pharmacist-led and comprehensive telehealth models, particularly in populations with poor baseline control, although comparisons of effect size across intervention categories should be interpreted cautiously because study designs, baseline HbA1c values, and effect measures varied. CGM-enabled personalization improved HbA1c in several studies and also improved CGM-derived glycemic metrics; in short-term interventions, improvements in peak glucose and vascular function were observed even when HbA1c did not change.

Risk of Bias

Risk of bias was assessed using RoB 2 for randomized trials and ROBINS-I for nonrandomized comparative studies. Study classification and risk-of-bias tool assignment were rechecked for consistency across the manuscript. Overall, most randomized trials were rated as having some concerns, with one study rated as high risk and one study rated as low risk (Table 5).

**Table 5:** Risk-of-bias (RoB) assessment for randomized trials using RoB 2, including cluster and crossover variants (n = 21). D1: Bias arising from the randomization process. D2: Bias due to deviations from intended interventions. D3: Bias due to missing outcome data. D4: Bias in measurement of the outcome. D5: Bias in the selection of the reported result. SC, some concerns; RoB 2-C, cluster randomized trial variant of RoB 2; RoB 2-XO, crossover trial variant of RoB 2

No.	Study (Reference)	Tool	D1	D2	D3	D4	D5	Overall risk
1	Lauffenburger et al. 2019 [18]	RoB 2	SC	SC	SC	Low	Low	Some concerns
2	Varming et al. 2019 [20]	RoB 2	SC	SC	SC	Low	SC	Some concerns
3	Frias et al. 2017 [23]	RoB 2-C	SC	SC	SC	Low	SC	Some concerns
4	Rein et al. 2022 [24]	RoB 2-XO	Low	SC	Low	Low	SC	Some concerns
5	Chang et al. 2025 [26]	RoB 2	SC	SC	SC	Low	SC	Some concerns
6	Slingerland et al. 2013 [27]	RoB 2-C	Low	SC	SC	Low	SC	Some concerns
7	Lee et al. 2018 [22]	RoB 2	Low	SC	SC	Low	SC	Some concerns
8	Javaid et al. 2019 [28]	RoB 2	SC	High	High	Low	SC	High risk
9	Ramallo-Fariña et al. 2020 [29]	RoB 2-C	SC	SC	SC	Low	SC	Some concerns
10	Griauzde et al. 2022 [30]	RoB 2	SC	SC	SC	Low	SC	Some concerns
11	Lee et al. 2022 [31]	RoB 2	SC	SC	SC	Low	SC	Some concerns
12	Crowley et al. 2022 [32]	RoB 2	SC	SC	Low	Low	SC	Some concerns
13	Martens et al. 2025 [34]	RoB 2	SC	SC	SC	Low	SC	Some concerns
14	Halalau et al. 2022 [35]	RoB 2	Low	SC	SC	Low	SC	Some concerns
15	Hangaard et al. 2025 [37]	RoB 2	SC	SC	SC	Low	SC	Some concerns
16	Caballero Mateos et al. 2025 [38]	RoB 2	SC	SC	SC	Low	SC	Some concerns
17	Bersch-Ferreira et al. 2024 [39]	RoB 2	SC	SC	SC	Low	SC	Some concerns
18	Romadlon et al. 2025 [41]	RoB 2	SC	SC	SC	Low	SC	Some concerns
19	Kulzer et al. 2018 [42]	RoB 2-C	SC	SC	SC	Low	SC	Some concerns
20	Shields et al. 2023 [43]	RoB 2-XO	Low	Low	SC	Low	Low	Low
21	Cox et al. 2020 [44]	RoB 2	SC	SC	SC	Low	SC	Some concerns

Summary of Quality Findings

Most studies were rated as having some concerns overall. This pattern is common in behavioral and digital health interventions, where blinding of participants and personnel is often not feasible because of the nature of the intervention. Notably, D4, bias in outcome measurement, was rated as low risk across nearly all studies because HbA1c is an objective laboratory measure and is therefore less vulnerable to observer bias, even in unblinded trials.

**Figure 2:**
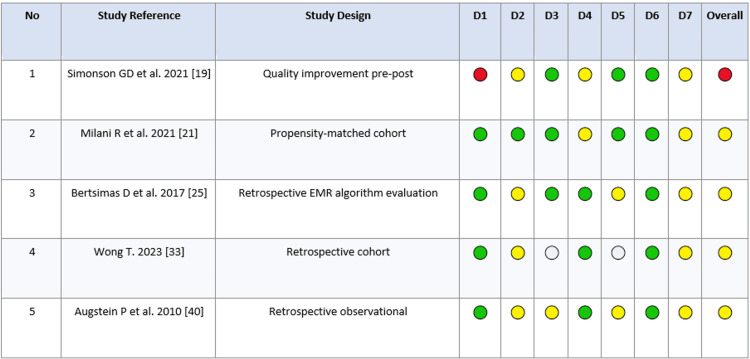
Risk-of-bias assessment of nonrandomized studies using a ROBINS-I-informed traffic light plot (n = 5). D1, confounding; D2, selection of participants; D3, classification of interventions; D4, deviations from intended interventions; D5, missing data; D6, measurement of outcomes; D7, selection of the reported result; Overall, overall risk of bias. Traffic light: 🟢 Low; 🟡 Moderate; 🔴 Serious; ⚪ No information

Among the nonrandomized studies, overall risk of bias generally ranged from moderate to serious, mainly due to confounding and selection-related bias. The uncontrolled quality improvement pre-post study was interpreted with additional caution because it lacked a concurrent comparator (Figure 2).

Discussion

Patterns of Effect Across Personalization Strategies

The included studies suggest that personalized medicine in T2DM is not a single, uniform intervention class. The effect depends on how personalization was defined, how intensively it was delivered, and whether patient-specific information was translated into clinical action. The most consistent benefit was seen when personalization combined risk stratification or objective monitoring with structured treatment adjustment. Other factors included behavioral reinforcement or medication optimization.

The relationship between care intensity and workflow-based personalization is illustrated in several key studies. These studies help demonstrate the different forms that personalization can take within routine healthcare settings.

In 2019, Lauffenburger et al. evaluated a scalable form of personalization using predictive analytics-based risk stratification to target support for insulin adherence [18]. Their findings showed improved insulin persistence with modest reductions in HbA1c in the subgroup with available laboratory follow-up [18]. This suggests that personalization at the level of outreach and care intensity may be feasible within routine health systems, especially when resources are limited. The modest HbA1c effect may reflect implementation constraints, such as incomplete uptake of pharmacist recommendations or variable patient exposure to the intervention. This interpretation aligns with concerns about the risk of bias profile assigned to this pragmatic trial [18].

Simonson et al. extended personalization into primary care workflow by using professional CGM to individualize medication and lifestyle decisions, with HbA1c improving by about 0.6 percentage points in a pre-post design [19]. However, this design limits causal inference because the absence of a concurrent comparator makes it difficult to distinguish intervention effects from regression to the mean, secular trends, and concurrent treatment intensification. This study is better interpreted as supportive feasibility evidence and an early signal of benefit rather than confirmatory evidence of efficacy [19].

Varming et al. examined a more relational form of personalization through patient-centered consultations focused on empowerment and adherence [20]. HbA1c decreased slightly in both study arms, without clear between-group separation [20]. This suggests that motivational or consultation-based personalization alone may have limited glycemic impact when not paired with frequent feedback, objective monitoring, or timely treatment adjustment. Empowerment remains important for long-term self-management, but its short-term glycemic effect may be modest without stronger mechanisms of action [20].

Telemonitoring and Digital Support

Milani et al. reported that home-based telemonitoring with connected glucose meters and pharmacist-supported management produced greater HbA1c improvement than matched usual care. This indicates a clinically meaningful but moderate effect [21]. However, because the evidence came from a propensity-matched cohort rather than randomized allocation, residual confounding remains possible. Patients who were more motivated or better resourced may have been more likely to engage with the intervention, which could have contributed to the observed benefit. These results support the practical value of telemonitoring and pharmacist-supported care, but they should be interpreted cautiously when estimating causal effect size and generalizability [21].

Lee et al. reported that tailored mobile coaching improved HbA1c and self-monitoring over six months, supporting the use of digitally tailored behavioral reinforcement in T2DM [22]. However, an open-label design, per-protocol analysis, and attrition concerns reduce certainty. The benefit direction is encouraging, though the effect size needs cautious interpretation [22].

Frias et al. evaluated digital medicines in a cluster-randomized pilot and reported a modest reduction in HbA1c, along with improvements in systolic blood pressure [23]. This pattern supports a plausible mechanism related to improved adherence and more responsive care delivery. However, cluster randomized pilot studies may be influenced by site-level differences in implementation fidelity and clinical culture, which limit certainty about the extent to which the observed effect is attributable to the intervention itself [23].

Personalized Nutrition and Algorithm-Guided Care

Rein et al. operationalized nutritional personalization using machine learning prediction of postprandial glycemic response, with improvement in CGM-derived metrics during the intervention phase and modest HbA1c reduction over follow-up [24]. These findings support the biological plausibility of tailoring diet to individual glycemic patterns, particularly in earlier disease stages. However, pilot designs provide limited evidence regarding long-term sustainability, scalability, and persistence of effect once intensive support and novelty diminish [24].

Bertsimas et al. approached personalization through the electronic medical record, estimating that algorithm-guided diabetes management could yield lower HbA1c than observed usual care [25]. Because the study was retrospective, causal inference remains limited by confounding by indication and differences in clinical follow-up that may not be fully captured even after statistical adjustment. These findings support the promise of algorithmic personalization as a systems-level tool, but prospective validation is required before firm conclusions can be drawn [25].

Chang et al. reframed personalization as timing, prescribing exercise relative to each individual’s peak hyperglycemia, and reported improvements in peak glucose and endothelial function [26]. This study highlights an important methodological point. When personalization targets glucose excursions rather than mean glycemia, CGM-derived metrics and vascular endpoints may detect benefit earlier and more sensitively than HbA1c [26].

Slingerland et al. provided a complementary systems-level perspective through stratified patient-centered care, where baseline HbA1c strata guided care intensity and cost-effectiveness, evaluated alongside clinical outcomes [27]. A greater reduction in HbA1c among participants with higher baseline values suggests that stratification may increase the impact of personalization by directing more intensive support to those most likely to benefit in absolute terms [27]. Together, these studies indicate that interpretation of personalized interventions should account for both the delivery level and the alignment between the intervention mechanism and the selected outcome [24,25,26,27].

Pharmacist-Led and Multicomponent Interventions

Javaid et al. reported substantial improvement in HbA1c in a pharmacist-led primary care management model [28]. However, the magnitude of the effect should be interpreted cautiously, as attrition and open-label care increase the risk of performance bias and differential follow-up. The direction of effect is clinically relevant, particularly in poorly controlled populations, but the certainty of the estimate remains limited [28].

Ramallo-Fariña et al. tested internet-based multicomponent interventions targeting both patients and professionals, including decision support, and observed a modest reduction in HbA1c [29]. This finding is consistent with the broader pattern in digital health, where effects may be small at the individual level but still meaningful at the population level if implementation is scalable and affordable. The average effect may mask greater benefit in selected higher-risk subgroups, although that possibility cannot be confirmed without more detailed subgroup reporting [29].

Griauzde et al. combined CGM with low-carbohydrate coaching in a pragmatic, randomized quality-improvement program and observed greater HbA1c improvement in high-risk participants compared with usual care [30]. This supports the interpretation that personalization is more effective when objective glucose data are paired with a structured behavioral prescription. The findings also suggest that targeted implementation in higher-risk subgroups may be more efficient than universal deployment [30].

Lee et al. reported an early HbA1c advantage at 12 weeks with an EMR-integrated mobile self-care application, but this benefit was not maintained at 26 weeks [31]. This time course suggests that early gains may attenuate when engagement declines, adaptive tailoring weakens, or background treatments change, reducing between-group differences. The study emphasizes the importance of sustained integration of personalized tools into clinical workflow if durable benefit is to be achieved [31].

Crowley et al. compared comprehensive telehealth with telemonitoring plus care coordination and demonstrated a clinically meaningful HbA1c advantage for the more comprehensive model over 12 months [32]. This finding indicates that telehealth should not be treated as a single exposure category. Greater benefit depends on whether the intervention includes medication management, structured behavioral support, and proactive follow-up rather than simple data transmission alone [32].

Wong reported a real-world association between personal CGM with clinician interpretation and improved HbA1c in insulin-treated outpatients [33]. Although this observational evidence aligns with results from randomized telehealth and coaching studies, interpretation remains limited by selection bias. In routine care, access to CGM is not random and may correlate with motivation, clinician attention, and social or economic support. These factors may inflate the apparent effect [33].

CGM-Enabled Self-Management and Adaptive Personalization

Martens et al. demonstrated that CGM-guided self-care to inform food choices reduced time above range and lowered HbA1c over six months in non-insulin-treated adults [34]. This supports the conclusion that CGM-based personalization is more likely to be effective when data are translated into explicit decisions about food intake and self-management rather than displayed passively. The added value of digital layers, such as app-based logging, may vary by context and user burden [34].

Halalau et al. reported one of the largest HbA1c improvements in the review within a pharmacist-managed clinic model [35]. This reinforces the broader pharmacist-led signal and suggests that higher-touch, personalized care, including barrier identification and medication optimization, may yield a greater absolute benefit, particularly when baseline HbA1c is high. Although part of the improvement may reflect intensified pharmacotherapy follow-up, this does not reduce the practical relevance of the model if it is deliverable in routine care [35].

Personalization may also be more effective earlier in the disease course. Li et al. reported that an internet-based management strategy for young adults with newly diagnosed diabetes was associated with better glycemic outcomes and higher remission rates over 12 months [36]. However, because the available summary provided limited design details, interpretation should remain cautious, and the findings should be viewed as promising rather than definitive [36].

Hangaard et al. shifted attention toward CGM-derived endpoints and reported meaningful improvement in time in range with telemonitoring compared with standard care among insulin-treated patients [37]. This is important because apparent inconsistency across the evidence base may partly reflect differences in outcome selection. Some interventions may improve daily glycemic exposure patterns and time in range before detectable separation in HbA1c occurs [37].

Caballero Mateos et al. evaluated digital education support in adults initiating glucagon-like peptide-1 (GLP-1) receptor agonists and found greater HbA1c improvement with the personalized digital program [38]. This context is clinically relevant because personalization may have its greatest incremental value during major treatment transitions, when adherence, side-effect management, and behavioral adaptation are evolving. These data suggest that the timing of personalization may be as important as its content [38].

Bersch-Ferreira et al. provided an important counterbalance, as their multicomponent nutritional strategy did not produce a statistically significant difference in HbA1c at six months despite a randomized, multicenter design [39]. This null result helps define the field's limits. Nutrition-focused interventions may fail to outperform usual care when personalization depth is limited, adherence declines, contamination occurs, or medication changes in both groups dominate glycemic outcomes. Differences in intervention granularity, objective feedback, and sustainability of support likely explain some of the heterogeneity across nutrition-related studies [39].

Decision Support, Messaging, and Precision Prescribing

Augstein et al. assessed the implementation of personalized decision support in routine care and observed modest improvements in HbA1c [40]. However, observational implementation studies remain vulnerable to confounding because better outcomes may reflect more engaged clinics and clinicians rather than the software itself. These findings support feasibility and potential utility, but not strong causal inference [40].

Romadlon et al. tested personalized diabetes text messaging with or without peer support and reported meaningful reductions in HbA1c, with large remission estimates and wide uncertainty [41]. The low cost and scalability of tailored messaging make this approach attractive, but the effect size warrants careful interpretation, as small sample sizes, baseline imbalance, and sensitivity to outcome definition may have influenced the results [41].

Kulzer et al. evaluated integrated personalized diabetes management using structured SMBG profiles and interpretation in a cluster randomized framework and found a small but statistically significant reduction in HbA1c at 12 months [42]. This study helps calibrate expectations because broad primary care personalization programs may yield modest average effects that are still worthwhile if scalable and cost-efficient [42].

Shields et al., through TriMaster, provided some of the strongest evidence in this review that measurable patient characteristics can modify medication response and guide actionable treatment selection [43]. Unlike many interventions that personalize education or monitoring, TriMaster addresses the precision medicine question directly at the level of drug choice. This suggests that stratified prescribing may represent one of the highest leverage forms of personalization in T2DM care [43].

Cox et al. demonstrated that CGM-guided individualized lifestyle and medication adjustments produced substantial improvements in HbA1c compared with routine care [44]. This finding integrates several consistent themes across the review. Personalization appears most effective when objective data are linked to a structured action plan and supported by clinical accountability. However, small sample sizes and variation in implementation intensity remain important constraints on certainty [44].

Integrated Synthesis and Clinical Implications

Across this evidence base, the most defensible conclusion is that personalization does not operate as a single class effect. Benefit appears to depend on three recurring elements. The first is targeting, particularly in individuals with higher baseline HbA1c or higher predicted risk. The second is objective feedback through CGM or telemonitoring. The third is the presence of an action pathway, such as pharmacist medication optimization, comprehensive telehealth, or decision support, that alters prescribing. Apparent contradictions across studies are therefore expected because interventions differed in intensity, comparator strength, engagement duration, and whether outcomes emphasized HbA1c or CGM-derived exposure metrics.

For clinical implementation, the strongest support comes from models that convert patient-specific data into actionable clinical decisions, particularly in individuals with poor glycemic control and during periods of therapeutic change. The evidence also suggests that higher-intensity, better-integrated models outperform lower-touch or purely informational approaches. For research, priorities include harmonized outcome sets that combine HbA1c with CGM metrics and patient-reported outcomes, longer follow-up to assess durability, more transparent reporting of engagement and co-interventions, and pragmatic randomized evaluations in real-world settings. Multicenter cluster randomized trials comparing CGM-informed decision support with protocolized treatment intensification at therapy escalation would be particularly valuable. Implementation-focused research, including mixed methods process evaluation and cost-effectiveness analysis, is also needed to identify the minimum effective dose of personalization, barriers to sustained engagement, and workflow requirements for scalable adoption.

Limitations of the Evidence Base and This Review

Several limitations should be considered when interpreting this review. First, the included interventions were highly heterogeneous in content, intensity, comparator, and duration, which limited direct comparability and precluded a single unified interpretation of effect size. Second, outcome reporting was not uniform across studies. While HbA1c was the most common endpoint, several interventions were more closely aligned with CGM-derived outcomes, such as time in range or peak glucose, which may explain some apparent inconsistency. Third, the certainty of evidence varied substantially across designs. Although randomized trials comprised the majority of included studies, some evidence came from observational, retrospective, or quality-improvement designs, which are more vulnerable to confounding, selection bias, and limited causal inference. Fourth, several trials raised concerns about open-label implementation, attrition, or selective reporting, which should temper the interpretation of the magnitude of the benefit. Finally, this review synthesized studies that evaluated multiple forms of personalization, including digital support, pharmacist-led care, CGM-based self-management, structured messaging, nutritional tailoring, and precision prescribing. While this broad scope improves conceptual understanding of the field, it also increases clinical and methodological heterogeneity and may reduce the precision of any single overarching conclusion.

## Conclusions

Personalized medicine interventions for T2DM were frequently associated with improved glycemic control, but their effectiveness depended on how personalization was operationalized. Approaches that translate patient-specific data into structured treatment decisions, especially within multidisciplinary or pharmacist-led models and among individuals with poor baseline control, appeared to offer the greatest clinical value. In contrast, low-intensity or purely informational personalization yields more variable results. Future research should prioritize pragmatic evaluations that integrate personalized decision pathways into routine care, use harmonized glycemic and patient-centered outcomes, and assess long-term sustainability to clarify where personalization is most likely to provide meaningful benefit in real-world diabetes management.

## Appendices

Appendix 

**Table 6:** Full search strategy across databases Reference lists of included studies and relevant reviews were hand searched to identify additional eligible articles. Duplicate records were removed before screening. Where database specific subject headings were available, these were combined with free text keywords to maximize sensitivity.

Database	Platform	Full final search string	Execution limits
PubMed or MEDLINE	PubMed	((Diabetes Mellitus, Type 2[Mesh] OR type 2 diabetes[tiab] OR T2DM[tiab]) AND (personalized[tiab] OR precision[tiab] OR individualized[tiab] OR tailored[tiab] OR stratified[tiab] OR patient centered[tiab] OR patient centered care[tiab] OR patient activation[tiab]) AND (Telemedicine[Mesh] OR telemedicine[tiab] OR telemonitoring[tiab] OR digital health[tiab] OR mHealth[tiab] OR app[tiab] OR apps[tiab] OR decision support[tiab] OR electronic health record*[tiab] OR EHR[tiab] OR CGM[tiab] OR continuous glucose monitoring[tiab] OR flash glucose monitoring[tiab]) AND (Hemoglobin A, Glycosylated[Mesh] OR HbA1c[tiab] OR glycemic control[tiab] OR glycaemic[tiab]))	Humans, English, Adults, publication years 2005 to 2025
Embase	Embase	('type 2 diabetes mellitus'/exp OR 'type 2 diabetes':ti,ab,kw OR t2dm:ti,ab,kw) AND ('personalized medicine'/exp OR personalized:ti,ab,kw OR precision:ti,ab,kw OR individualized:ti,ab,kw OR tailored:ti,ab,kw OR stratified:ti,ab,kw OR patient centered:ti,ab,kw OR patient activation:ti,ab,kw) AND ('telemedicine'/exp OR telemedicine:ti,ab,kw OR telemonitoring:ti,ab,kw OR digital health:ti,ab,kw OR mhealth:ti,ab,kw OR app:ti,ab,kw OR apps:ti,ab,kw OR decision support system/exp OR decision support:ti,ab,kw OR electronic health record:ti,ab,kw OR ehr:ti,ab,kw OR cgm:ti,ab,kw OR continuous glucose monitoring:ti,ab,kw OR flash glucose monitoring:ti,ab,kw) AND ('glycosylated hemoglobin'/exp OR hba1c:ti,ab,kw OR glycemic control:ti,ab,kw OR glycaemic:ti,ab,kw)	English, Adults, publication years 2005 to 2025
Cochrane CENTRAL	Cochrane Library	([mh Diabetes Mellitus, Type 2] OR type 2 diabetes:ti,ab,kw OR T2DM:ti,ab,kw) AND (personalized:ti,ab,kw OR precision:ti,ab,kw OR individualized:ti,ab,kw OR tailored:ti,ab,kw OR stratified:ti,ab,kw OR patient centered:ti,ab,kw OR patient activation:ti,ab,kw) AND (telemedicine:ti,ab,kw OR telemonitoring:ti,ab,kw OR digital health:ti,ab,kw OR mHealth:ti,ab,kw OR app:ti,ab,kw OR apps:ti,ab,kw OR decision support:ti,ab,kw OR EHR:ti,ab,kw OR CGM:ti,ab,kw OR continuous glucose monitoring:ti,ab,kw OR flash glucose monitoring:ti,ab,kw) AND (HbA1c:ti,ab,kw OR glycemic control:ti,ab,kw OR glycaemic:ti,ab,kw)	No database level filters applied at execution; year, language, and age restrictions applied during screening
CINAHL	EBSCOhost	((MH Diabetes Mellitus, Type 2+) OR TI type 2 diabetes OR AB type 2 diabetes OR TI T2DM OR AB T2DM) AND (TI personalized OR AB personalized OR TI precision OR AB precision OR TI individualized OR AB individualized OR TI tailored OR AB tailored OR TI stratified OR AB stratified OR TI patient centered OR AB patient centered OR TI patient activation OR AB patient activation) AND (TI telemedicine OR AB telemedicine OR TI telemonitoring OR AB telemonitoring OR TI digital health OR AB digital health OR TI mHealth OR AB mHealth OR TI app OR AB app OR TI apps OR AB apps OR TI CGM OR AB CGM OR TI continuous glucose monitoring OR AB continuous glucose monitoring OR TI flash glucose monitoring OR AB flash glucose monitoring OR TI decision support OR AB decision support) AND (TI HbA1c OR AB HbA1c OR TI glycemic control OR AB glycemic control OR TI glycaemic OR AB glycaemic)	English, Adults, publication years 2005 to 2025
Scopus	Scopus	(TITLE-ABS-KEY(type 2 diabetes OR T2DM) AND TITLE-ABS-KEY(personalized OR precision OR individualized OR tailored OR stratified OR patient centered OR patient activation) AND TITLE-ABS-KEY(telemedicine OR telemonitoring OR digital health OR mhealth OR app OR apps OR decision support OR electronic health record* OR EHR OR CGM OR continuous glucose monitoring OR flash glucose monitoring) AND TITLE-ABS-KEY(HbA1c OR glycemic control OR glycaemic)) AND PUBYEAR > 2004 AND PUBYEAR < 2026 AND LIMIT-TO(LANGUAGE, English)	English, publication years 2005 to 2025
Web of Science	Web of Science Core Collection	TS=((type 2 diabetes OR T2DM) AND (personalized OR precision OR individualized OR tailored OR stratified OR patient centered OR patient activation) AND (telemedicine OR telemonitoring OR digital health OR mhealth OR app OR apps OR decision support OR electronic health record* OR EHR OR CGM OR continuous glucose monitoring OR flash glucose monitoring) AND (HbA1c OR glycemic control OR glycaemic))	English, publication years 2005 to 2025, articles and early access where available
Google Scholar	Google Scholar	type 2 diabetes AND (personalized OR tailored OR precision OR CGM OR telehealth OR telemonitoring OR digital health) AND (HbA1c OR glycemic control)	English, publication years 2005 to 2025, first 200 results screened by relevance, plus citation chasing
